# Supplementary material for: Enhanced neuroimaging with a calcium sensor in ex-vivo Drosophila melanogaster brains using closed-loop adaptive optics light-sheet fluorescence microscopy
Source: J Biomed Opt. 2023 Jun 16;28(6):066501. doi: 10.1117/1.JBO.28.6.066501 (PMC10275380; doi:10.1117/1.JBO.28.6.066501)
Supplement: Supplementary file 1 [file JBO_028_066501_SD001.pdf]

Supplemental Materials: Enhanced neuroimaging with a calcium sensor in *ex vivo Drosophila melanogaster* brains using closed-loop adaptive optics light-sheet fluorescence microscopy

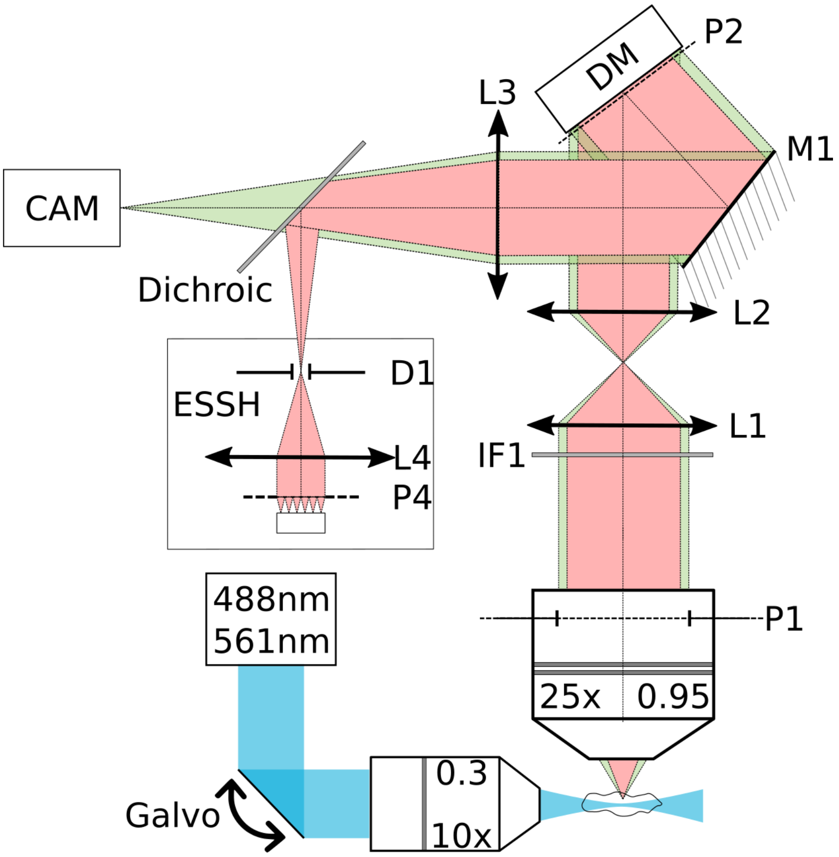

Fig S1 AO-LSFM detailed setup for functional imaging from Fig.1, red and green beams are of different sizes for representation purpose only, P#: Pupil plane, L#: achromatic doublet converging lens DM: Deformable Mirror, M#: mirror, D1: iris, excitation objectives and detection objectives NA's and magnification are respectively (0.3,10X and 0.95,25X)

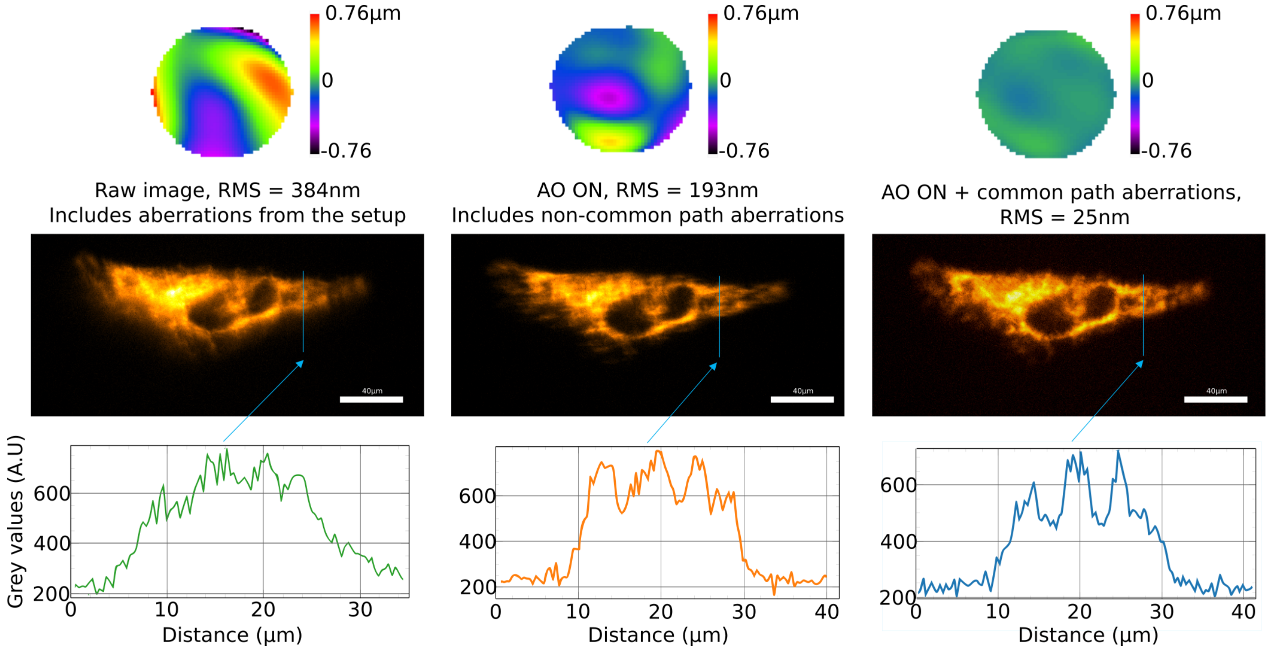

Fig S2 A living HeLa cell labeled with MitoTrackerGreen (Thermofisher) imaged using the same optical setup with an extra epifluorescence arm with a 488nm laser beam. The thickness of the cell is comparable to the thickness of the sheet in the light-sheet configuration and acts as a guide plane for the ESSH analyzer. Images on the scientific camera (left) with a static correction of the aberrations of the set-up from the objective to the wavefront analyzer (center) with a static correction of the set-up aberrations, including those of the non-common path to the imaging camera (right) with full correction of both set-up and sample induced aberrations.

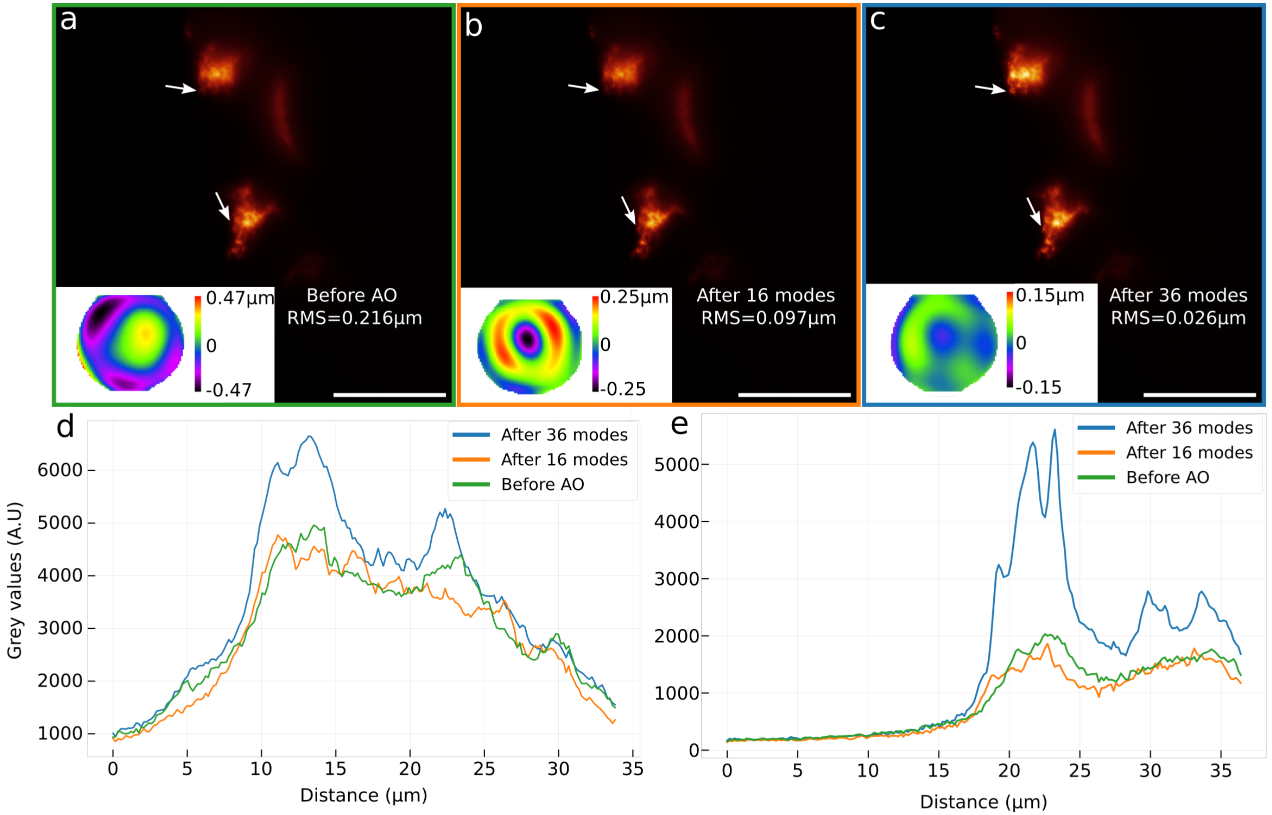

Fig S3 Full field of view of Fig.2 (top) projections of sleeping neurons at 60-80µm in depth, dual labeled with GFP/ChRFP, in an *ex vivo* adult *Drosophila* brain, with associated wavefronts and profiles in two separated areas (a) without correction, (b) with 16 DM modes for correction and (c) with 36 DM modes for correction. Line profiles in (d) correspond to centered white arrows and line profiles in (e) to top white arrows. The wavefront is estimated on a 130x130µm^2 FOV located on the projections in the center of the image. Scale bar: 100µm
